# Evaluation of Extended Interval Dosing Aminoglycosides in the Morbidly Obese Population

**DOI:** 10.1155/2013/194389

**Published:** 2013-08-19

**Authors:** Ashley L. Ross, Jennifer L. Tharp, Gerald R. Hobbs, Richard McKnight, Aaron Cumpston

**Affiliations:** ^1^Department of Pharmacy, Jewish Hospital, 200 Abraham Flexner Way, Louisville, KY 40202, USA; ^2^Department of Pharmacy, Johnson City Medical Center, 400 North State of Franklin Road, Johnson City, TN 37604, USA; ^3^Department of Statistics, West Virginia University, P.O. Box 9190, Morgantown, WV 26506, USA; ^4^Department of Pharmacy, West Virginia University Healthcare, 1 Medical Center Drive, Morgantown, WV 26506, USA; ^5^Osborn Hematopoietic Malignancy and Transplantation Program, Mary Babb Randolph Cancer Center, West Virginia University, Morgantown, WV 26506, USA

## Abstract

Aminoglycoside dosing has been studied in the obese population, typically recommending an adjusted weight utilizing a 40% dosing weight correction factor (IBW + 0.4 × (TBW–IBW)). These studies included limited numbers of morbidly obese patients and were not done in the era of extended interval aminoglycoside dosing. Here, we report a retrospective evaluation of morbidly obese patients receiving gentamicin or tobramycin at our hospital. The objective of this study was to evaluate the accuracy of the commonly recommended adjusted weight for weight-based dosing. There were 31 morbidly obese patients who received gentamicin or tobramycin 5–7 mg/kg every 24 hours using a 40% dosing weight correction factor. Our institution utilizes 16-hour postdose concentrations to monitor extended interval aminoglycosides. Twenty-two of the 31 patients (71%) achieved an appropriate serum drug concentration. Four patients (13%) were found to be supratherapeutic and 5 patients (16%) subtherapeutic. The only variable that correlated with supratherapeutic levels was older age (*P* = 0.0378). Our study helps to validate the current dosing weight correction factor (40%) in the morbidly obese population. We recommend caution when dosing aminoglycosides in morbidly obese patients who are of older age.

## 1. Background

The World Health Organization (WHO) estimated that 500 million people worldwide were obese in 2008 and that number would increase to 700 million by 2015 [1]. In 2011, all 50 states in the United States estimated that 20% or more of their adult population was obese (body mass index ≥ 30 kg/m^2^); 12 out of the 50 states estimated that more than 30% of their adult population was in this category [2]. Not only the prevalence of obesity is rapidly increasing, but the weight of obese patients continues to rise with five percent of Americans now being considered morbidly obese (body mass index ≥ 40 kg/m^2^) [3].

Several clinical studies, case reports, and review articles have described that pharmacokinetic properties are altered in obese patients compared to ideal body weight (IBW) patients [4–15]. There are four main issues that merit discussion when evaluating pharmacokinetic alterations in obesity: absorption, distribution, metabolism, and elimination. The absorption of most drugs is thought to be relatively unaffected by obesity [16]. However, studies in obese patients have shown an altered volume of distribution due to their increased blood flow and cardiac output, changes in protein binding, and organ mass [16, 17]. The metabolism of drugs in this particular population is poorly understood, but may be altered [3]. Elimination, on the other hand, has been shown to be affected. Obese patients are thought to have an increased glomerular filtration rate, theoretically leading to increased renal elimination of medications. This increased clearance is most likely multifactorial. Obese patients have been shown to have larger glomerular planar surface area compared to nonobese controls [18]. These patients also exhibit higher cardiac output, hyperperfusion to the kidney, and glomerular hypertension [3, 17–20]. A trial by Bauer et al. described obese patients having an altered glomerular filtration rate and increased aminoglycoside drug clearance [20].

Although there are multiple studies documenting the use of aminoglycosides in obese patients, there is a lack of data specifically in the morbidly obese population, which has been identified as greater than or equal to 190% of IBW or a body mass index (BMI) of greater than or equal to 40 kg/m^2^ [20, 21]. A dosing weight correction factor (DWCF) is commonly utilized in the obese population for weight-based dosing to estimate a percentage of the excess weight (total body weight minus IBW) that should be applied to the dosing scheme. The study by Bauer et al. included 30 morbidly obese patients who were greater than or equal to 190% of their IBW receiving tobramycin, gentamicin, or amikacin. However, traditional or conventional dosing methods were utilized. A DWCF of 38–58% was recommended [20]. Traynor et al. reported on 524 obese patients (greater than 125% of IBW) receiving traditional doses of either tobramycin or gentamicin. The average weight of all participants was only 150% of IBW in this study, and a DWCF of 43% was recommended [21]. A recent study by Pai et al. found that lean body weight may yield more consistent estimates of volume of distribution in the obese patients [22]. Limited data exist in the morbidly obese population, and none of these studies were done in the era of extended interval aminoglycosides. 

## 2. Objectives

The primary objective of this study was to assess whether previously published DWCF result in appropriate aminoglycoside drug levels in the morbidly obese population utilizing extended interval dosing. Secondary objectives were to determine if there are patient-specific characteristics in the morbidly obese patient that may impact therapeutic level achievement.

## 3. Methods

Patients receiving extended interval gentamicin or tobramycin for any infectious cause between December 2005 and December 2008 at West Virginia University Healthcare (WVUH) were retrospectively evaluated. Patients were included in the analysis if gentamicin or tobramycin extended interval dosing was used, a 16 hour serum drug concentration was obtained, and patients were 190% or greater than their IBW. IBW was calculated as 2.3x inches over 5 feet in height, plus 45.5 for females and 50 for males. Patients were excluded from the analysis if serum creatinine was greater than 1.5 mg/dL, data was unavailable for estimation of IBW or creatinine clearance (CrCl), they were less than 18 years of age, dialysis patients, obstetric patients, or patients with cystic fibrosis. Patient information was collected from a computerized physician order entry system, an electronic patient database, nursing and physician notes, and billing records. This study was approved by the Institutional Review Board of WVUH.

Data collected on each patient included age, gender, weight, height, serum creatinine, drug, dose, serum drug concentrations, and timing of sample collection. In addition, serum creatinine was documented for five days after the start of therapy, if available, to identify fluctuations in renal function. An IBW and an adjusted body weight (ABW), using a DWCF of 40%, were calculated for each patient. To calculate an estimated CrCl, the Salazar-Corcoran formula was utilized [23–26]. Drug concentrations were drawn after the first dose of aminoglycosides.

Patients were further evaluated if they were given a dose of 5–7 mg/kg every 24 hours using a 40% DWCF. This subset of patients was assessed for accuracy of achieving therapeutic levels. Levels were deemed therapeutic if the level drawn 16 hours after dose was less than or equal to 2 mcg/mL but still detectable (lower limit of detection 0.5 mcg/mL). This is the standard therapeutic approach at our institution based on the expected duration of the postantibiotic effect of extended interval aminoglycosides lasting approximately 6–8 hours. Therefore, concentrations at 16 hours that were undetectable would be considered subtherapeutic. Concentrations greater than 2 mcg/mL at 16 hours after dose would be excessive, based on previously published nomograms [27, 28].

## 4. Statistical Analysis

Statistical analysis was done on all patient variables. The response was categorical. When predictor was categorical, a contingency chi-square test was utilized. Whereas, when a predictor was evaluated, logistic regression analysis was done. A *P* value of less than 0.05 was interpreted as statistically significant.

## 5. Results

Forty patients met the study inclusion and exclusion criteria. Of the 40 patients included in the analysis, 31 patients were dosed within the range of 5–7 mg/kg of ABW using a 40% DWCF. These patients were analyzed to determine appropriateness of serum concentrations achieved as well as to determine if any patient characteristics had an impact on level achievement. The patient demographics on these 31 patients can be found in Table 1. Of the 31 patients dosed with 5–7 mg/kg every 24 hours, 22 patients (71%) had a therapeutic concentration, 4 patients (13%) had a supratherapeutic level, and 5 patients (16%) had a subtherapeutic level.

**Figure 1:**
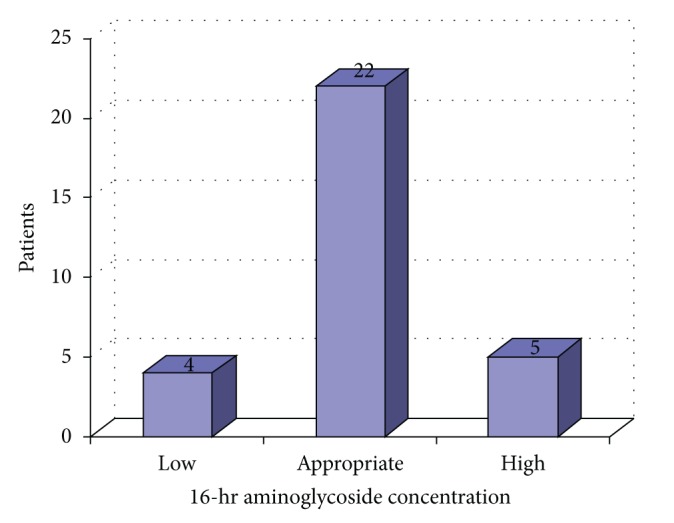
Aminoglycoside serum concentrations.

Of the 16 patients that received gentamicin, 14 patients (88%) had therapeutic drug levels, whereas only 8 out of the 15 patients (53%) who received tobramycin had therapeutic drug levels (*P* = 0.326). Serum concentration achievement and patient demographics can be found in Figure 1 and Table 2, respectively. Older age did correlate with higher aminoglycoside serum concentrations (*P* = 0.0378). There was no significant difference using multiple logistic regression analysis between serum concentrations and patient weight, gender, height, or estimated creatinine clearance. Two out of the thirty-one patients (6.5%) had renal toxicities, defined as a serum creatinine greater than or equal to two times their baseline level. Renal toxicities occurred in one patient in both the supratherapeutic concentration and therapeutic concentration groups.

## 6. Discussion

Our data report morbidly obese patients receiving gentamicin or tobramycin at a dose of 5–7 mg/kg every 24 hours, based on the patient's ABW and using a 40% DWCF. Serum drug concentrations were found appropriate in 71% of patients. There was also a similar number of patients who were either supratherapeutic or subtherapeutic. This observation is a valuable addition to current knowledge of aminoglycoside use in obesity and expands the available literature, specifically in the morbidly obese patient. It is the first report, to our knowledge, utilizing extended interval dosing in this patient population.

Extended interval aminoglycosides are included in various protocols within our hospital, with dosing schemes ranging from 5 to 7 mg/kg every 24 hours. The recommended therapeutic drug monitoring includes a serum concentration drawn 16 hours after the dose, with the desired level being less than 2 mcg/mL, but detectable, to help ensure drug clearance and a suitable postantibiotic time period. At our institution, we have decided to use a 16-hour interval as the time point to evaluate drug concentrations with extended interval aminoglycosides. However, we also realize that many institutions may utilize a different time point or a two-level monitoring method.

Maximum (*C*_max⁡_) and minimum (*C*_min⁡_) serum drug concentrations in all patients could not be calculated with a single serum concentration. Although not preferred based on potential pharmacokinetic discrepancies, we estimated renal function and population kinetics to calculate an estimated mean *C*_max⁡_ of 18.3 mg/dL + 2.7 (14.5–22.3 mg/dL) and *C*_min⁡_ of 0.06 mg/dL + 0.09 (0–0.32 mg/dL). Actual pharmacokinetic calculations could be done on three patients due to the availability of a second serum concentration checked within the dosing interval (Table 3). The mean *C*_max⁡_ and *C*_min⁡_ for these three patients were 19.7 mg/dL + 2.9 and 0.5 mg/dL + 0.08, respectively.

In this study, the Salazar-Corcoran formula was used to estimate each patient's CrCl. Although the Cockcroft-Gault formula is the predominant methodology to estimate CrCl by clinicians, the Salazar-Corcoran formula has been shown through retrospective trials to be the most precise when dealing with obese patients [3, 23, 24]. In addition, the Salazar-Corcoran formula was developed using an obese rat model, and its effectiveness has been validated through human clinical trials [3, 24–26]. The modifications of diet in renal disease (MDRD) and the chronic kidney disease-epidemiology (CKD-EPI) methods are alternative formulas that may be helpful to estimate glomerular filtration rate in the future, but at this time, more studies need to be done to validate their use in the obese and morbidly obese populations [3, 22].

We chose to define our patients as morbidly obese using IBW instead of BMI. We made this decision under the assumption that most clinicians treating adult patients are more accustomed to drug dosing based on ideal, adjusted, or total body weight. By using IBW to define morbidly obese patients, we anticipate that our data can be utilized by clinicians more easily and with more confidence that it relates to their daily practice.

It is not surprising to find a correlation with increasing age and higher aminoglycoside serum concentrations. This is most likely related to declining renal function resulting in less aminoglycoside clearance in the older population. Caution should be taken with the older patient and possible consideration for some empiric dose reductions based on age and renal function.

There are many limitations of this study. It is a retrospective analysis with a relatively small sample size. However, it is the only study currently in the literature that evaluates extended interval gentamicin or tobramycin dosing in the morbidly obese population. Although we evaluated gentamicin and tobramycin, we did not include morbidly obese patients who were administered extended interval doses of amikacin. We excluded these patients because of the low number of patients on amikacin at our hospital during the study period.

## 7. Conclusion 

Obese patients have unique pharmacokinetics, which can make drug dosing a difficult task. A 40% DWCF seems to remain accurate in morbidly obese patients, even in the era of extended interval aminoglycoside dosing. We caution practitioners on the dosing of older patients, as they are prone to supratherapeutic levels, even with the estimation of good renal function.

## Figures and Tables

**Table 1:** Patient demographics.

Patient-specific parameters (*n* = 31)	
Patients receiving gentamicin	52%
Gender	68% females
Mean age	51 years ± 13.3
Mean weight	127.7 kg ± 24.5
Mean weight above IBW	212% ± 24.7
Mean aminoglycoside dosing	5.9 mg/kg ± 0.9
Mean serum creatinine	0.86 mg/dL ± 0.2
Mean CrCl	132.3 mL/min ± 43.2

**Table 2:** Patient-specific data based on achievement level.

	Age	Percent above IBW	CrCl (mL/min)
High level *N* = 4	61.5 ± 12.3	210.4% ± 27.1	107.3 ± 53.1
Appropriate level *N* = 22	50.5 ± 12.4	213.7% ± 25.9	137.1 ± 44.0
Low level *N* = 5	43.4 ± 14.7	206.0% ± 20.7	131.2 ± 31.2

**Table 3:** Patient pharmacokinetic parameters when two concentrations were available.

	Elimination rate (H^−1^)	Volume of distribution (L/kg)	Half-life (Hrs)	*C*_max⁡_ (mg/dL)	*C*_min⁡_ (mg/dL)
Patient 1	0.149	0.23	4.6	19.6	0.6
Patient 2	0.156	0.29	4.4	16.8	0.4
Patient 3	0.158	0.29	4.4	22.6	0.5
